# Tandem alternative polyadenylation events of genes in non-eosinophilic nasal polyp tissue identified by high-throughput sequencing analysis

**DOI:** 10.3892/ijmm.2014.1734

**Published:** 2014-04-07

**Authors:** PENG TIAN, JIE LI, XIANG LIU, YUXI LI, MEIHENG CHEN, YUN MA, YI QING ZHENG, YONGGUI FU, HUA ZOU

**Affiliations:** 1 Department of Otolaryngology-Head and Neck Surgery, Sun Yat-sen Memorial Hospital, Sun Yat-sen University, Guangzhou 510120, P.R. China; 2 State Key Laboratory for Biocontrol, Guangdong Province Key Laboratory of Pharmaceutical Functional Genes, Department of Biochemistry, College of Life Science, Sun Yat-sen University, Higher Education Mega Center, Guangzhou 510006, P.R. China

**Keywords:** chronic rhinosinusitis, nasal polyps, alternative polyadenylation site, 3′ untranslated region

## Abstract

Nasal polyps (NP) is highly associated with the disorder of immune cells. Alternative polyadenylation (APA) produces mRNA isoforms with different length of 3′-untranslated region (UTR) and regulates gene expression. It has been proven that this APA-mediated regulation of 3′UTR length is an immune-associated phenomenon. The aim of this study was to investigate the genome-wide alternative tandem 3′UTR length switching events in non-eosinophilic nasal polyp tissue. Thirteen patients diagnosed as having non-eosinophilic nasal polyps were included in this study. Nasal polyp tissue and control mucosa were collected during surgery. The 3′ end library of cDNA was constructed. The recovered libraries were sequenced with second sequencing technology, and the sequencing data were analyzed by an in-house bioinformatics pipeline. Tandem 3′UTR length switching between samples was detected by a test of linear trend alternative to independence. We found a significant alteration in the tandem 3′UTR length in 1,920 genes in nasal polyp samples. Functional annotation results showed that several gene ontology (GO) terms were enriched in the list of genes with switched APA sites, including regulation of transcription, macromolecule catabolic localization and mRNA processing. The results suggested that APA-mediated alternative 3′UTR regulation plays an important role in the post-transcriptional regulation of gene expression in non-eosinophilic nasal polyps.

## Introduction

Nasal polyps (NP) is a chronic inflammatory disease of the mucosa of the paranasal sinuses and nasal cavities, which are often present in conjunction with chronic rhinosinusitis (CRS). It has a great adverse impact on the quality of life and daily activities of its sufferers and often requires surgical intervention for treatment. Histological studies have demonstrated that nasal polyp tissue contains large quantities of lymphocytes, eosinophils, neutrophils as well as plasma cells, indicating this is a disease resulting from disorders of immunological and inflammatory response (1). Although factors influencing gene expression in nasal polyp tissue have been widely studied over the last two decades, the ultimate factors inducing the development of nasal polyps remain uncertain (2).

Precise cleavage and polyadenylation during transcription is a fundamental part of eukaryotic gene expression. The complexity of the eukaryotic transcriptome is greatly expanded by the use of alternative polyadenylation (APA) sites. APA sites located on the last exon can produce 3′-untranslated region (3′UTR) with different lengths (tandem 3′UTR) leading to altered post-transcriptional regulation of gene expression (3). Two-thirds of all human genes undergo APA (4). Moreover, widespread APA has been shown in multiple organisms and systems. Given the known regulatory role of 3′UTRs in mRNA localization, stability and translation, APA alteration is bound to impact these functions, and defects of APA lead to human diseases (5). With the exception of several diseases resulting from the APA disorder, such as oculopharyngeal muscular dystrophy (OPMD) (6), thalassemias (7), and thrombophilia (8), the emerging cases concerning the connection between polyadenylation dysfunction and pathological situations (including cancer, immunity and inflammation and viral infection) have been identified. Studies have shown that mRNAs with shorter 3′UTRs and alternatively spliced isoforms resulting from usage of more proximal APA sites are characteristic of activated immune, neuronal and cancer cells (9–11).

Typically, APA sites have been analyzed either using northern blot or by microarray technology through a ‘subtractive’ analysis approach. Next-generation sequencing (NGS) technology has largely improved the potential to study APA, and a number of NGS-based strategies have been devised to directly profile APA patterns. Compared with conventional microarrays, the NGS technology allows direct measurement of usage of each APA instead of comparing probes within different regions of a 3′UTR. Notably, this type of method provides a comprehensive landscape of APA site switching (including known and unknown APA sites) instead of only known APA sites.

In a previous study concerning eosinophilic nasal polyp, we identified a large set of genes with 3′UTRs that varied in length from eosinophilic CRSwNP patients (12). Several Gene Ontology terms, including transcription regulation, cell cycle, apoptosis, were enriched in the list of genes with switched APA sites (12). In the present study, using a genome-wide 3′UTR sequencing method based on NGS technology, we analyzed APA switching events in nasal polyp tissue derived from Chinese patients with non-eosinophilic chronic rhinosinusitis with nasal polyps (NE-CRSwNP). The results showed that, in NE-CRSwNP, genes exhibiting 3′UTR switching were enriched for functional groups such as transcription, protein transport and nucleic acid-binding and processing factor, suggesting distinct pathogenesis from E-CRSwNP. Furthermore, we validated our findings and demonstrated the prevalence of APA site changing events of *CD163*, *GRB2*, *BCAP29* and *TAX1BP* genes in an additional 10 patients.

## Materials and methods

### Ethics

This study was undertaken according to the institutional approval from the local research ethical committee (the Internal Review and the Ethics Boards of the Sun Yat-sen Memorial Hospital, Sun Yat-sen University). Informed written consent was provided by all participants.

### Subjects and samples

Thirteen patients presenting to the Sun Yat-sen Memorial Hospital of Sun Yat-sen University who met the diagnostic criteria for CRSwNP based on clinical parameters including absence of asthma and allergic rhinitis were eligible for the study. All the patients were confirmed as having non-eosinophilic NPs (NE-NPs) according to the histological appearance, which showed a lack of eosinophilic infiltration, as determined by an experienced pathologist. All the patients failed to respond to medical treatments and therefore underwent functional endoscopic sinus surgery. Those patients with antrochoanal polyps, cystic fibrosis, fungal sinusitis, primary ciliary dyskinesia or systemic diseases were excluded. The clinical information of the participants is listed in Table I.

Fresh tissue samples (including nasal polyp samples and matched uncinate process mucosa) were obtained from 13 patients with NE-CRSwNPs during endoscopic sinus surgery. The samples were sectioned and submerged in RNAlater^®^ solution (Qiagen, Valentia, CA, USA) to avoid RNA degradation as per the manufacturer’s instructions. All the tissues treated were maintained at −20°C for subsequent extraction.

### RNA extraction and quality control

Total RNA of the samples was extracted with TRIzol reagent (Invitrogen, Carlsbad, CA, USA) following the manufacturer’s instructions. The extracted total RNA quantity was determined with a Nanodrop ND-1000 spectrophotometer (Isogen). In addition, the quality was analyzed with electrophoresis in a 1% agarose gel, and the value of A260/A280 (ratios between 1.9 and 2.1 were considered acceptable). The extracted RNA was digested with RNase-free DNase (Toyobo, Osaka, Japan).

### Construction of 3′end sequencing libraries

The 3′end libraries were prepared as previously described (13,14). Briefly, total RNA was randomly fragmented by heating. The fragmented total RNA was reverse transcribed with a modified anchored oligo(d)T primer containing an Illumina adaptor sequence. Concurrently, a 5′template-switching adaptor tagged with Illumina adaptors was added. The reaction was conducted in an improved reverse transcription reaction mixture using the Super-Script II reverse transcriptase enzyme (Invitrogen Life Technologies, Karlsruhe, Germany). ds-cDNA was synthesized by PCR amplification with known sequencing primers and Platinum® Taq DNA Polymerase High Fidelity (Invitrogen). Size selection was conducted by performing PAGE separation, excision, and gel extraction. The final pooled libraries were then generated and sequenced from the 3′end with an Illumina Solexa GA IIx (Illumina, San Diego, CA, USA).

### Bioinformatics methods

#### Analysis of 3′UTR sequencing data

The 3′UTR sequencing data were processed as described previously (13,14). Briefly, the raw reads were trimmed, filtered, and aligned to the human genome (hg19) using Bowtie (version 0.12.5) (15). Total read numbers from each sample were normalized to cancel bias induced by different samples. In addition, the uniquely mapped reads were clustered to define poly(A) cleavage sites. The poly(A) cleavage sites with two or more reads were used for subsequent analysis. Two or multiple poly(A) site clusters within the 3′UTR of a gene were defined as tandem alterative poly(A) (APA) sites. Our study focused only on this type of APA sites.

#### APA switching analysis in usage of alternative poly(A) sites

The test of linear trend alternative to independence was performed as described previously in order to detect the genes with significant 3′UTR length changes between samples (16). In this study, we designated the matched control mucosa as 1 and the nasal polyp tissue as 2. Therefore, a negative significant Pearson correlation r indicated that nasal polyp tissue contains shorter tandem 3′UTR length than the control.

#### DAVID analysis of 3′UTR length changed genes

Functional annotation of the detected 3′UTR-lengthened and -shortened genes between the nasal polyp tissue and control mucosa was performed with the DAVID Bioinformatics Resources (http://david.abcc.ncifcrf.gov/) (17). We searched for significantly enriched biological process, GO terms, and pathways against a background model of all transcripts found in all the samples.

### Quantitative RT-PCR (qRT-PCR) validation

qRT-PCR experiments were conducted to validate the APA patterns of genes that exhibited significant and obvious APA switches in the 3′end sequencing experiment in non-eosinophilic nasal polyp tissue. The target sites of the qRT-PCR primers were located in the common and extensive regions of the transcript. The qRT-PCR was performed using the Light Cycler 480 instrument (Roche Biochemicals, Indianapolis, IN, USA) with Thunderbird™ SYBR qPCR Mix (Toyobo, Osaka, Japan) according to the manufacturer’s instructions. The expression ratios of the common/shortened region to the extensive/lengthened region (cUTR/eUTR) were obtained by calculating ΔΔCt values for each gene by normalizing the extensive set against the constitutive one. Significantly differential usage of poly(A) sites of genes between samples was detected by the Student’s t-test at a significant level of 0.05.

## Results

### Clinical manifestations

**Figure 1:**
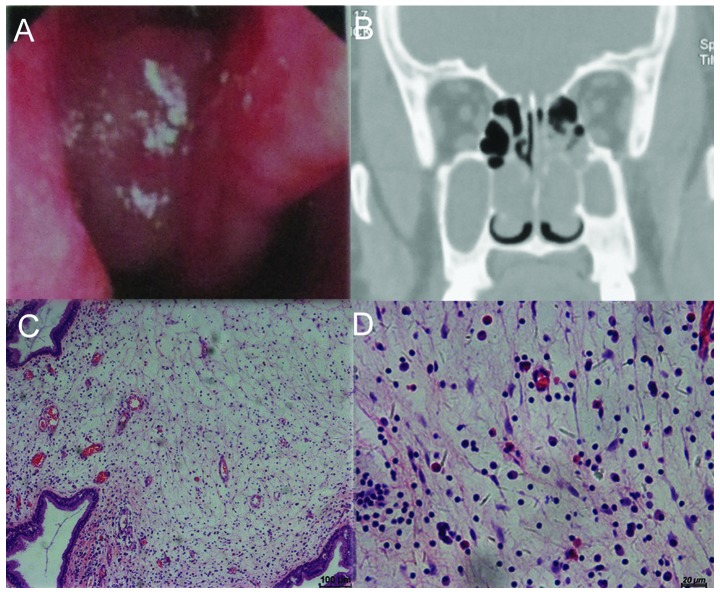
Histological analysis showing the types of nasal polyps in Chinese patients. (A) Nasal endoscopic findings of nasal polyps. (B) Representative paranasal sinus computed tomography (CT) findings of nasal polyps. (C) Histological appearance (magnification, ×100). (D) Histological appearance (magnification, ×400).

Thirteen patients who were diagnosed as NE-CRSwNPs were selected for this study. The patients had negative prick tests and an absence of allergies in their personal histories. Typically semitransparent nasal lesions appeared in the nasal cavity (Fig. 1A). Representative CT image is shown in Fig. 1B. Histologically, the nasal polyps showed prominent infiltration of inflammatory cells (mostly lymphocytes and plasma cells instead of esoinphils) (Fig. 1C and D). The clinical characteristics of all thirteen patients are summarized in Table I. The small sample size was selected as this study was an exploratory study and elementary analysis.

### A comprehensive analysis of 3′ends of mRNAs

**Figure 2:**
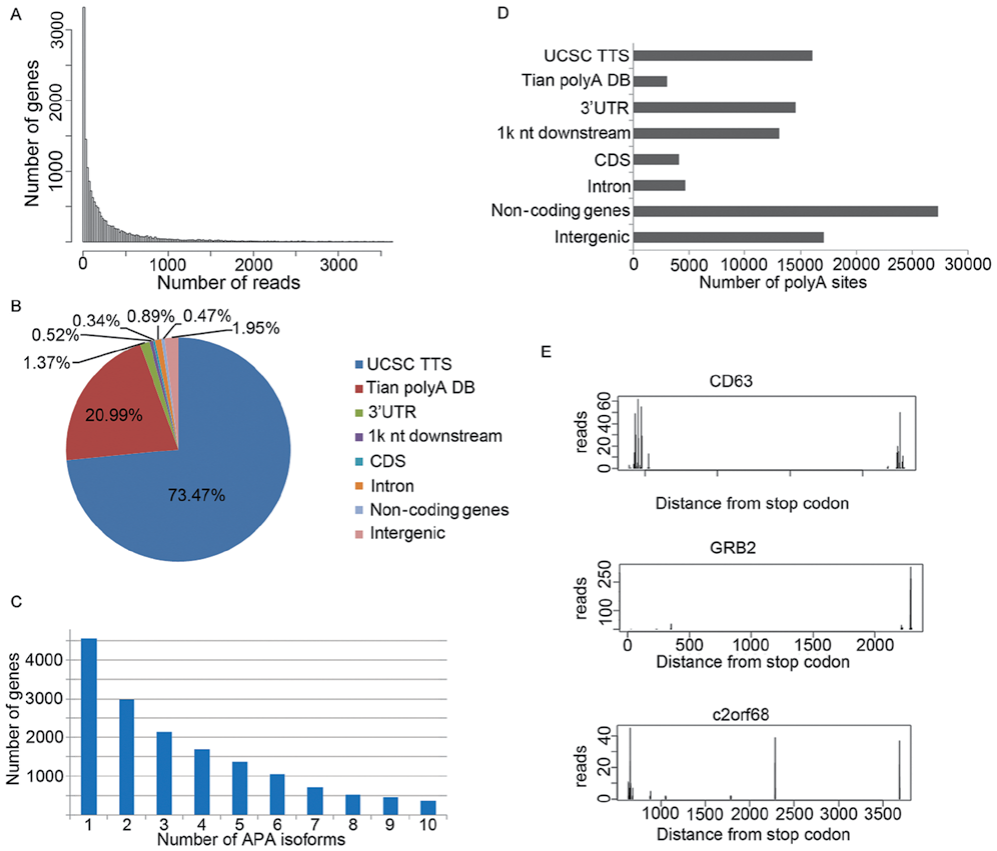
The characteristics of the sequencing data. (A) The genomic locations of the poly(A) sites in all genes. (B) A histogram of the number of reads for the UCSC canonical gene. (C) The genomic locations of reads that were uniquely mapped to the nuclear genome after internal priming filtering. (D) A bar graph showing the number of genes with a different number of poly(A) sites, each number ‘n’ on the x-axis means n or more APA isoforms were detected. (E) Examples of tandem-UTR genes with two, three, and seven 3′UTR isoforms.

The first aim was to directly and comprehensively profile the APA sites of two human non-esoinophilic NP samples and their uncinate process mucosa. For this purpose, we used the method of sequencing APA sites (SAPAS) with next-generation sequencing technology and a bioinformatic pipeline to analyze the sequencing data as described previously (13). In all, we obtained about 102 million raw reads with lengths of 75 bp. After excluding the reads without modified anchor oligo d(T), we obtained 89.5 million (87.5%) reads, of which ~59.7 (58.4%) million reads uniquely were assigned to the human nucleus genome (hg19). When the reads with internal priming were filtered, 45.7 million reads (44.8%) could be used to directly infer transcript cleavage sites. In addition, 17,921 UCSC canonical genes were sampled by at least one read, accounting for 60% of all canonical genes. A statistical summary of the data is shown in Table II. The distribution of the number of all reads is shown in Fig. 2A.

The majority of the filtered reads (94.5%) produced in this study were assigned to known poly(A) sites listed in the UCSC transcripts end database and Tian’s database, and an additional 1.4 and 0.5% reads were mapped to the 3′UTR and 1 kb downstream from the UCSC canonical genes, respectively (Fig. 2B) (4).

In total, we identified of 99,866 cleavage sites, representing 17,042 genes, in the combined samples. 11,825 (72.2% of the 17,042 genes) genes were detected with two or more cleavage sites (Fig. 2C; with three examples presented in Fig. 2D). We found ~19.1% of these sites in the UCSC and Tian’s databases and another 27.4, 14.6 and 13.1% of the poly(A) sites in the introns, 3′UTRs and CDSs from the UCSC canonical genes, respectively (Fig. 2E).

### Differential usage of alternative 3′UTRs between nasal polyp and paired mucosa

**Figure 3:**
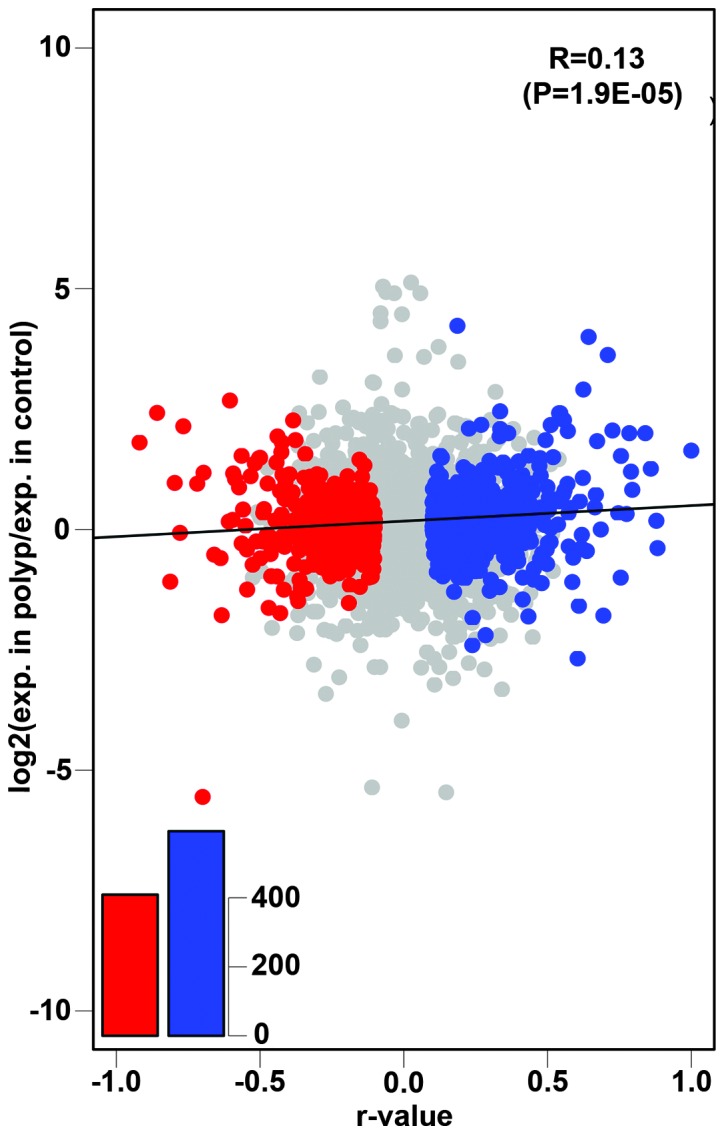
The representative example shows an association between APA site switching and gene expression levels of nasal polyp tissue and normal mucosa. A larger positive r-value shows that nasal polyp samples are prone to use longer tandem 3′UTRs. Genes with significant switching to longer (blue) or shorter (red) tandem 3′UTRs in nasal polyp samples (FDR=0.01; Materials and methods) are colored. The y-axis shows the logarithm of the expression levels of genes from the nasal polyp sample corresponding to the nasal mucosal sample.

Results of recent studies demonstrated that 3′UTR switching events were associated with the progression of colorectal cancer (18), embryonic development or organogenesis (14,19), and immune regulation of lymphoblastoid cells (20). In this study, we compared the tandem 3′UTR length of non-eosinophilic nasal polyp tissue to matched uncinate process mucosa by analysis of the distribution of obtained reads over different cleavage sites for a gene with the linear trend alternative to independence test. We observed significant alteration in the tandem 3′UTR length in 1,920 genes [false discovery rate (FDR)=0.01] including 1,029 genes which were inclined to use longer 3′UTR isoforms and 891 genes which were prone to use shorter 3′UTR isoforms in nasal polyp samples (Fig. 3). The findings indicated that there appeared to be an equal representation of switching to longer and shorter 3′UTR isoforms. This result was inconsistent with the tendency of 3′UTR switching in cancer cells.

Variation in 3′UTR length may lead to the gain or loss of regulatory elements, such as miRNA binding sites, and then impact the stability of mRNA. To determine whether there was a correlation between the alteration in 3′UTR length and changes in gene expression levels, the extent of APA changes with changes in mRNA abundance was compared. Of note, we did not observe a global correlation between the 3′UTR length and gene expression (Fig. 3). This result was in accordance with previous reports (13,21). The reason for this result, however, remains to be determined.

To validate the sequencing data, four APA-switched genes were selected and qRT-PCR was performed in sequencing samples. This method allows us direct and accurate measurement of the relative abundance of each mRNA isoforms. As to the four selected genes (*CD163*, *GRB2*, *BCAP29* and *TAXBP1*) which exhibited extreme 3′UTR length differences relative to controls, our results showed the same trend as the sequencing findings, and therefore confirmed our findings in sequencing samples (Table III).

### Functional enrichment of genes exhibiting switched APA

To evaluate the roles of genes exhibiting 3′UTR length variation, we searched for enriched functional groups among the genes exhibiting altered APA using the web-accessible DAVID program (false discovery rate: 10%, maximum P<0.01). The enrichment results are shown in Tables IV and V. In general, 3′UTR-shortened genes were enriched for the categories of regulation of transcription, protein targeting and transport, macromolecule catabolic localization and RNA splicing. The 3′UTR-lengthened genes were enriched for categories such as protein localization, mRNA metabolic process and mRNA processing.

Of note, these APA-changing gene products were rarely found as the constituents of non-membrane organelles (such as ribosomes) and seldom participated in biosynthetic processes. For assessment of cellular localization, the products of these genes were enriched in intracellular processes and were not found in the extracellular region. These results suggested that the genes actively involved in intracellular processes, and not the genes involved in basic cell structure or routine biological processes, were mainly targeted by tandem APA in the development of nasal polyps.

To evaluate the roles of the regulations of tandem 3′UTRs in signaling cascades, we assigned the APA sites-switching genes to KEGG pathways. We found that no pathways were significantly enriched in the APA-switching genes in nasal polyp tissue.

### APA patterns suggesting novel mechanism mediating development of nasal polyps

**Figure 4:**
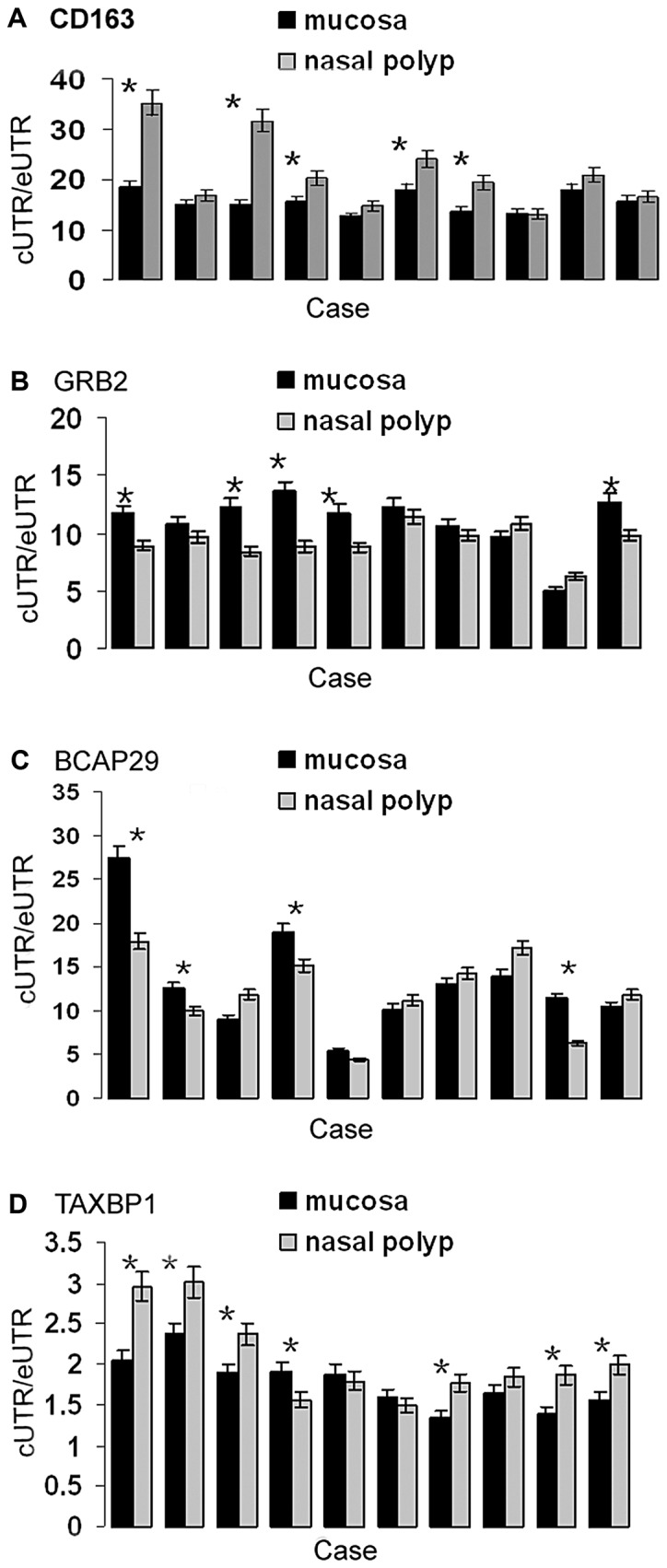
qRT-PCR analyses in 10 additional patients suffering from NE-CRSwNP. (A) CD163. (B) GRB2. (C) BCAP29. (D) TAX1BP. cUTR/eUTR, common UTR/extensive UTR, the expression ratios of the shortened region to the lengthened region. ^*^P<0.05.

To assess the prevalence of APA site-changing events, we used qRT-PCR technique to measure APA patterns of the above four selected genes in additional 10 samples (including nasal polyp tissue and matched control mucosa). We found that the genes *CD163*, *GRB2*, *BCAP29* and *TAX1BP* exhibited the same APA trends as the sequencing samples in 4, 6, 5 and 4 patients, respectively, and the difference was statistically significant (P<0.05) (Fig. 4). The results of the larger cohort subjects with NE-NPs also indicated that this 3′UTR length shift mediated by the selection of the APA sites may be an important gene expression regulation mechanism in nasal polyp development.

## Discussion

In this study, we performed a global direct measurement of 3′ends of mRNAs of nasal polyp samples and matched inflammatory mucosa. We profiled APA-switching patterns of mRNAs in non-eosinophilic nasal polyp tissue and identified APA-switched genes that were involved in transcription regulation, protein targeting and transport, protein localization, RNA splicing and mRNA processing. As the development of nasal polyps involves cell proliferation and immune response, our data suggest that many of the APA changes described in this study may contribute to these aspects of nasal polyps. This provided a novel perspective for the pathogenesis of nasal polyp.

The global gene set enrichment analyses indicates the importance of 3′UTR switching in non-eosinophilic NPs. We compared the GO terms of eosinophilic NPs with that of non-eosinophilic NPs and found that although the two nasal polyp samples showed altered 3′UTRs in many mRNAs, different enrichments of GO terms and pathways were identified in the APA-switched genes. For example, the APA-switched genes in eosinophilic NPs are mainly enriched on apoptosis (12), while the 3′UTR switched genes in non-eosinophilic NPs are mainly enriched on transcription and proliferation. The differences may be explained by different histological and immunological features of non-eosinophilic compared with eosinophilic NPs.

The selection in APA sites undoubtedly leads to loss or gain of important sequences in the untranslated regions at the 3′end of mRNA transcripts and then influences the fate of mRNA and thus proteosynthesis. The changes in APA patterns of mRNA transcripts in nasal polyp tissue revealed by our study undoubtedly reflect pathological changes that contribute to the pathogenesis of NPs. Further investigation of the genes with variable 3′UTR length may identify some of the genes that may be targeted in the treatment and prevention of NPs, for example, the subsequent 4 APA-switched genes.

One of the genes revealed by this study, *TAX1BP1*, produces human T-cell leukemia virus type I binding protein 1, which interacts with other important molecules such as A20 and TRAF6, which participate in inflammatory response processes (22). The *CD163* gene encodes a multiligand scavenger receptor protein exclusively expressed by monocytes and macrophages. It functions as an innate immune sensor for bacteria and inducer of local inflammation (23). Recent studies have revealed its important roles for immune defense of the host against *S. aureus* infections (24).

The third gene, *GRB2*, encodes for a gene critical in the signal transduction pathway. It is ubiquitously expressed and binds the fibroblast growth factor receptor (FGFR) family of receptor tyrosine kinases (RTKs), which are key regulators of many cell processes, including proliferation, cell-cycle control, migration and differentiation (25). Of note, the shift of APA patterns of GRB2 is still largely unknown in the field of immune response of nasal mucosa.

Another gene of the identified genes is B-cell receptor-associated protein 29 (*BCAP29*). This gene is known to have several 3′UTR isoforms of mRNAs, derived from multiple PAS sites (26). Although this *BCAP29* gene has been associated with the WNT pathway, the *BCAP29* gene itself is still largely unknown in the field of immunity and inflammation. Notably, additional 3′UTR data produced by our study from eosinophilic nasal polyp tissue also showed a highly significant APA shift, suggesting a more general role for this gene in nasal polyp.

In summary, results of the present study showed that, the global analyses of mRNA alternative polyadenylation have demonstrated that RNA alternative polyadenylation is more prevalent than previously thought since APA regulates the usage of the 3′UTR and, thus, the cis-elements of mRNAs in non-eosinophilic nasal polyp tissue. Our results suggest that APA-mediated alternative 3′UTR regulation may play an important role in the post-transcriptional regulation of gene expression and pathogenesis of non-eosinophilic nasal polyp tissue and that the distinct mechanism from eosinophilic nasal polyps leads to the development of non-eosinophilic nasal polyps. Future studies should elucidate the role of APA regulation in driving the non-eosinophilic immune response through the genes mentioned as well as other genes.

## Figures and Tables

**Table I:** Data of inflammatory cell frequency in nasal polyp tissue of non-eosinophilic CRSwNP patients.

Case	Age (yrs.)/Gender	Lymphocytes (%)	Eosinophil (%)
1	23/Male	48.6	4.9
2	30/Male	54.4	4.6
3	35/Female	58.3	4.5
4	47/Male	93.6	4.3
5	68/Female	59.2	0
6	31/Female	56.2	4.8
7	45/Male	68.3	4.6
8	35/Male	45.3	2
9	36/Female	34.4	3.5
10	41/Female	60.3	4.5
11	45/Male	58.3	3.7
12	20/Female	37.2	4.1
13	43/Female	93.6	3.5

All the NPs were non-eosinophilic.

**Table II:** Summary statistics for solexa sequencing and mapping to genome (hg19).

Items	Case 55C	Case 55P	Case 36C	Case 36P	Combined
Raw reads	30,415,461	29,508,427	20,287,449	22,109,878	102,321,215
Qualified reads	27,625,126	27,287,883	16,827,740	17,809,279	89,550,028 (87.5%)
Mapped to genome	25,514,090	25,966,853	14,800,273	15,205,149	81,486,365 (79.3%)
Uniquely mapped to genome	19,239,132	19,756,273	10,240,035	10,469,750	59,705,190 (58.4%)
Mapped to nuclear genome	18,739,483	19,285,503	10,022,826	10,268,225	58,316,037 (57.0%)
After IP filter	17,911,620	18,568,301	9,309,362	9,526,473	45,789,283 (44.8%)
Genes sampled by reads	17,312	17,143	16,469	16,652	17,921
Cleavage clusters	84,155	81,992	58,762	60,183	99,866
Known polyA sites sampled	26,783	26,508	22,441	22,354	27,643
Putative novel polyA sites	57,372	55,484	36,321	37,829	72,223
Genes sampled by cleavage clusters	16,598	16,497	15,596	15,634	17,042

IP, internal priming; C, control mucosa; P, polyp tissue.

**Table III:** Validation of 3′UTR switching in nasal polyp compared with control mucosa using qRT-PCR

	Illumina sequencing data			qRT-PCR			
			
UCSC ID	3′UTR	Pearson’s r	FDR	3′UTR	P-ER	C-ER	P-value
uc011jzo.1	Shortened	−0.12009	4.82E-03	Shortened	31.71780	15.16862	<0.001
uc003vej.2	Lengthened	0.39509	2.85E-08	Lengthened	8.820350	13.59187	<0.001
uc001qsz.3	Lengthened	0.226097	4.00E-05	Lengthened	9.961412	12.59653	<0.001
uc002jnx.3	Shortened	−0.182239	1.42E-08	Shortened	2.375458	1.896878	<0.001

ER, expression ratios of the common region to the extended region; P, polyp tissue; C, control tissue. Pearson’s r, a larger positive/negative value indicates that longer/shorter tandem UTRs are prone to be used in the nasal polyp.

**Table IV:** Enrichment results of genes with significantly shorter 3′UTR isoforms involved in various GO functional categories.

Categories	Count	P-value	Benjamini
GOTERM_BP_FAT			
Transcription	151	1.70E-07	4.20E-04
Regulation of transcription	167	3.50E-05	1.30E-02
Intracellular protein transport	40	4.50E-06	5.70E-03
Intracellular transport	58	1.10E-05	8.90E-03
Protein targeting	27	1.40E-05	8.90E-03
Protein transport	64	1.60E-05	8.30E-03
Establishment of protein localization	64	2.20E-05	9.40E-03
Cellular protein localization	40	4.00E-05	1.30E-02
Cellular macromolecule localization	40	4.70E-05	1.30E-02
Protein localization	69	6.90E-05	1.50E-02
Modification-dependent macromolecule catabolic process	50	6.60E-05	1.70E-02
Modification-dependent protein catabolic process	50	6.60E-05	1.70E-02
Protein catabolic process	53	6.90E-05	1.60E-02
Proteolysis involved in cellular protein catabolic process	51	1.00E-04	2.00E-02
Cellular protein catabolic process	51	1.20E-04	2.10E-02
RNA splicing	29	2.60E-04	4.30E-02
GOTERM_CC_FAT			
Membrane-enclosed lumen	123	1.50E-05	6.60E-03
Organelle lumen	121	1.50E-05	3.40E-03
Nuclear lumen	101	1.50E-05	2.30E-03
Nucleoplasm part	49	1.60E-05	1.80E-03
Intracellular organelle lumen	118	2.30E-05	2.10E-03
Nucleoplasm	66	8.80E-05	6.50E-03
Spliceosome	17	3.50E-04	2.20E-02

**Table V:** Enrichment results of genes with significantly longer 3′UTR isoforms involved in various GO functional categories.

Categories	Count	P-value	Benjamini
GOTERM_BP_FAT			
Protein localization	82	3.70E-06	1.00E-02
Cellular macromolecule localization	47	5.00E-06	6.80E-03
Cellular protein localization	46	9.20E-06	8.40E-03
mRNA metabolic process	42	1.80E-05	1.20E-02
mRNA processing	55	2.50E-05	1.40E-02
Establishment of protein localization	70	4.30E-05	2.00E-02
Protein transport	69	5.90E-05	1.80E-02
GOTERM_CC_FAT			
Organelle lumen	138	1.80E-05	8.70E-03
Intracellular organelle lumen	135	2.30E-05	5.50E-03
Membrane-enclosed lumen	138	4.80E-05	7.50E-03
Nuclear lumen	111	1.10E-04	1.30E-02
Golgi apparatus	71	4.60E-04	4.30E-02
